# Patients With Super Obesity Do Not Perceive Themselves as Being at Higher Risk for a More Severe Course of COVID-19 Infection

**DOI:** 10.3389/fpsyt.2021.798662

**Published:** 2022-02-24

**Authors:** Norbert Schäffeler, Jacqueline Lohmiller, Isabelle Mack, Rami Archid, Stephan Zipfel, Andreas Stengel

**Affiliations:** ^1^Department of Psychosomatic Medicine and Psychotherapy, University Hospital Tübingen, Tübingen, Germany; ^2^Department of General, Visceral, and Transplant Surgery, University of Tuebingen, Tuebingen, Germany; ^3^Charité Center for Internal Medicine and Dermatology, Department for Psychosomatic Medicine, Charite-Universitätsmedizin Berlin, Corporate Member of Freie Universität Berlin, Humboldt-Universität zu Berlin and Berlin Institute of Health, Berlin, Germany

**Keywords:** obesity, body perception, desire for weight reduction, risk factors, risk patients

## Abstract

**Background:**

The coronavirus disease 2019 (COVID-19) pandemic has led to major health-related concerns in the population. Several risk factors for a severe course of COVID-19 disease have been identified, with obesity taking an important role. However, it is unclear whether this association is only known in the expert world or whether individuals also experience themselves as risk patients due to their obesity and whether the desire for weight reduction may also be associated with a hoped-for risk reduction. These questions were addressed in a cross-sectional study of patients who have presented to an obesity center in order to lose body weight.

**Methods:**

Patients (*n* = 155) of the obesity center were asked to complete an *ad hoc* questionnaire to assess whether the desire to lose weight is also associated with a hoped-for risk reduction with respect to COVID-19 disease during the middle of the pandemic in the period between October 2020 and April 2021. We additionally assessed their perceived general stress using the Perceived Stress Questionnaire (PSQ).

**Results:**

In our explorative study, overall worries correlated significantly with worries about contracting COVID-19 (*r* = 0.483, *p* < 0.001). There has been an association with concerns about severe COVID-19 progression and psychological distress from the COVID-19 pandemic (*r* = 0.543, *p* < 0.001). In addition, a correlation was found between persons who worry about contracting COVID-19 and feeling like an at-risk patient (*r* = 0.530, *p* < 0.001). Interestingly, the higher the BMI (>50 kg/m^2^), the lower were the worries in PSQ (ANOVA *p* = 0.046). However, COVID-19-related worry was nonetheless present in the higher BMI subgroups. The most intense worries were experienced by individuals with a BMI between 35 and 39 (PSQ worries 50.44), immediately followed by individuals with a BMI between 40 and 49 (PSQ worries 49.36).

**Discussion and Conclusion:**

An increased risk for a more severe course of COVID-19 infection is not generally perceived by obese individuals. In particular, individuals with very high BMI (>50)—although being at very high risk for a severe course of the COVID-19 disease—do not display increased worries, which might point toward heightened denial.

## Introduction

The coronavirus disease 2019 (COVID-19) was caused by a novel coronavirus called severe acute respiratory syndrome coronavirus 2 (SARS-CoV-2) (1). The mild to moderate form of COVID-19 illness is associated with cough, fever, and fatigue. However, if a severe course of COVID-19 disease is present, it can lead to pneumonia, respiratory failure, multiorgan failure, and ultimately death (1, 2). The number of global deaths associated with COVID-19 disease increased to 4.86 million confirmed deaths by mid-October 2021 (3). In light of the ongoing COVID-19 crisis, studies have been initiated worldwide to investigate the severity of the disease and health risk factors. Some studies have already been able to identify confirmed risk factors that may cause COVID-19 disease to be life-threatening (4). Some research manifested that elderly patients and patients with chronic pre-existing conditions such as obesity, cardiovascular disease, diabetes, cancer, and chronic respiratory and renal diseases are vulnerable to severe COVID-19 disease (5–7).

There is no clear evidence of an increased risk of contracting COVID-19 due to existing obesity. On the other hand, it has undoubtedly been identified that obesity must be considered an underlying risk factor for severe COVID-19 disease progression. A recent study in England showed a linear increase of risk of severe COVID-19 starting from BMI ≥23 (7–9). Obesity as an important risk factor for a severe COVID-19 course is characterized by increased hospital admissions, intensive care, and ventilator requirement with fatality (10). This risk liability even emerges independently of age, gender, and comorbidities (7, 9). Thus, there is the worrying increased risk for severe COVID-19 progression in obesity and other chronic diseases that are often concomitant and secondary to obesity. For subjects with obesity, the COVID-19 pandemic therefore entailed enormous challenges.

The lockdown certainly had the potential to promote weight gain through social isolation, an accompanying lack of physical activity, and the swelling of the stress gamut that often accompanies increased consumption of unhealthy foods (11–13). Both depression and anxiety disorders can be severe psychological comorbid diseases, which can be aggravated by the above-mentioned COVID 19 consequences and accordingly promote the risk of a severe COVID-19 disease course (11). We examined whether subjects with obesity perceive themselves as being at-risk patients regarding a COVID-19 infection, and if so, whether they are afraid of an infection or willing to reduce weight because of this risk. Ultimately, we investigated whether the desire for weight reduction of patients with obesity may also be related to a hoped-for risk reduction for a COVID-19 disease.

## Methods

### Procedure

We conducted an explorative cross-sectional online survey to assess whether the desire to lose weight is also associated with a hoped-for risk reduction with respect to COVID-19 disease course or progression. The study was carried out between October 2020 and April 2021 at the University Hospital in Tübingen. The patients who have presented to our obesity center in order to lose body weight have been examined at the psychosomatic outpatient clinic at the University Hospital in Tübingen from psychologists and psychosomatic doctors. All patients in the study period were offered the opportunity to participate in the survey. The patients, who had given their written consent after detailed information and explanation of the study, participated in the anonymous data collection. The questionnaire was handed out in digital form by email after each consultation. The study was approved by the local ethics committee (458/2020BO).

### Sample

A total of 155 patients (113 female, 42 male) participated in the survey. We had to exclude one female and one male participant because of invalid data regarding weight and height (final *n* = 153). The following analyses are based on the descriptive and psychometric data collected using the online questionnaire.

### Survey

In total, the questionnaire consisted of three subject areas:

- “patient characterization” with three nominally (patients' wish for obesity treatment, treatment received or current treatment, and month of appointment) and five metrically (age, sex, weight, height, and comorbidities) scaled items;
- “COVID-19-related concerns” with one nominally scaled item (COVID-19 infection in the past—yes, no) and nine items recorded on Likert scales (ranging from 0 = do not agree at all to 100 = agree fully—see Table 1); and
- the standardized questionnaire “Perceived Stress Questionnaire” (PSQ) (14). The “PSQ” encompassed 20 items recorded on a 4-point Likert scale with the categories “almost, never, sometimes, often, most of the time” and measures worries, tension, joy and demands on patients (15). Cronbach's alpha and split half reliability for subscales is >0.70.

**Table 1:** COVID-19-related concerns.

**Question**	**Average**
	**0 (strongly disagree)**
	**to 100 (strongly agree)**
**Anxiety and worries**	
(1) How much psychological distress do you feel as a result of the COVID 19 pandemic?	40.98 (SD 27.78)
(2) I am worried that I will get COVID-19 myself.	35.95 (SD 32.04)
(3) I am concerned that COVID-19 disease will take a severe course in me.	43.92 (SD 35.99)
(4) I am generally quick to worry or fret.	34.72 (SD 33.88)
(5) I have withdrawn socially because of my concern about contracting COVID-19.	33.64 (SD 34.74)
**Risk perception**	
(6) I feel like I am an at-risk patient because of my obesity.	48.48 (SD 39.27)
(7) Reducing the risk of getting severely ill with COVID-19 is an important motivation for me to reduce weight.	41.92 (SD 39.22)
**Control**	
(8) I have the feeling that the state and the relevant authorities have the situation regarding COVID-19 under control.	40.63 (SD 28.97)
(9) I feel sufficiently informed with regard to COVID-19.	59.66 (SD 32.70)

### Statistical Analyses

The analyses were performed using the statistical software IBM SPSS Statistics (version 27, IBM Corp, 2017). Sociodemographic characteristics were analyzed descriptively. After assessing normal distribution of data, differences were assessed using a *t*-test and χ^2^-test. In addition, ANOVAs were used to test whether BMI (grouped) had an impact on COVID-19-related worries or general worries. Spearman correlation analyses were performed to examine associations, e.g., between COVID-19-related concerns and PSQ concerns. Because of the explorative manner of this study, we did not perform a power analysis and did not adjust for multiple testing.

## Results

### Patient Characterization

The study population consisted of 153 patients (112 female, 41 male). The mean age was 43.0 years (range = 18–77 years, SD 12.8). BMI ranged from 31.2 to 61.7 kg/m^2^, average 45.4, SD 6.2. Two percent reported a BMI between 30 and 34.9 kg/m^2^, 19.6% BMI 35 to 39.9 kg/m^2^, 58.2% BMI 40 to 49.9 kg/m^2^, and 20.3% had a BMI >50 kg/m^2^ (Table 2); 77.1% reported one or more somatic comorbidities, and 31.4% reported psychological comorbidity (depression and/or anxiety disorder); 19.6% reported no comorbid diseases (Table 2). There has been no significant correlation between BMI and number of reported somatic as well as psychological comorbidities. Patients' desired treatment related to BMI groups shows an increase of desired bariatric treatment with higher BMI, while conservative treatment decreases [see Table 2, χ^2^ (3) = 20.55, *p* < 0.001].

**Table 2:** Patient characterization.

**Characteristic**			***n***	**%**
**Gender**				
Male			41	26.8
Female			112	73.2
**BMI group**				
BMI 30–34.9 kg/m^2^ (group 1)			3	2.0
BMI 35–39.9 kg/m^2^ (group 2)			30	19.6
BMI 40–49.9 kg/m^2^ (group 3)			89	58.2
BMI >50 kg/m^2^ (group 4)			31	20.3
**Comorbid diseases**				
Arterial hypertension			73	47.7
Hyperlipoproteinemia			28	18.3
Diabetes mellitus			30	19.6
COPD			3	2.0
Sleep apnea			44	28.8
Joint degeneration			73	47.7
Depression			43	28.1
Anxiety disorder			14	9.2
No comorbid diseases			30	19.6
**Desired treatment**				
	**Conservative treatment**		**Bariatric**	
	**(guided weight loss)**		**surgery**	
	***n***	**%**	***n***	**%**
BMI group 1 (*n* = 3)	0	0.0	2	66.7
BMI group 2 (*n* = 30)	20	66.7	15	50.0
BMI group 3 (*n* = 89)	28	31.5	78	87.6
BMI group 4 (*n* = 31)	3	9.7	29	93.5
			χ^2^ (3) = 20.55	*p* < 0.001

*N = 153 subjects. Desired treatment: patients could choose multiple options; therefore, here the number exceeds n = 153*.

### COVID-19-Related Concerns

A COVID-19 infection was reported by 3.3%. Patients' concerns related with COVID-19 have been moderate to medium. On average, patients stated that they feel like being an at-risk patient because of being obese with 48.5 on a scale from 0 to 100. Their motivation to reduce weight because of thereby reducing the risk of getting severely ill with COVID-19 has been 41.9 on average. Feeling sufficiently informed with regard to COVID-19 has been 59.7 on average (see Table 1).

**Figure 1:**
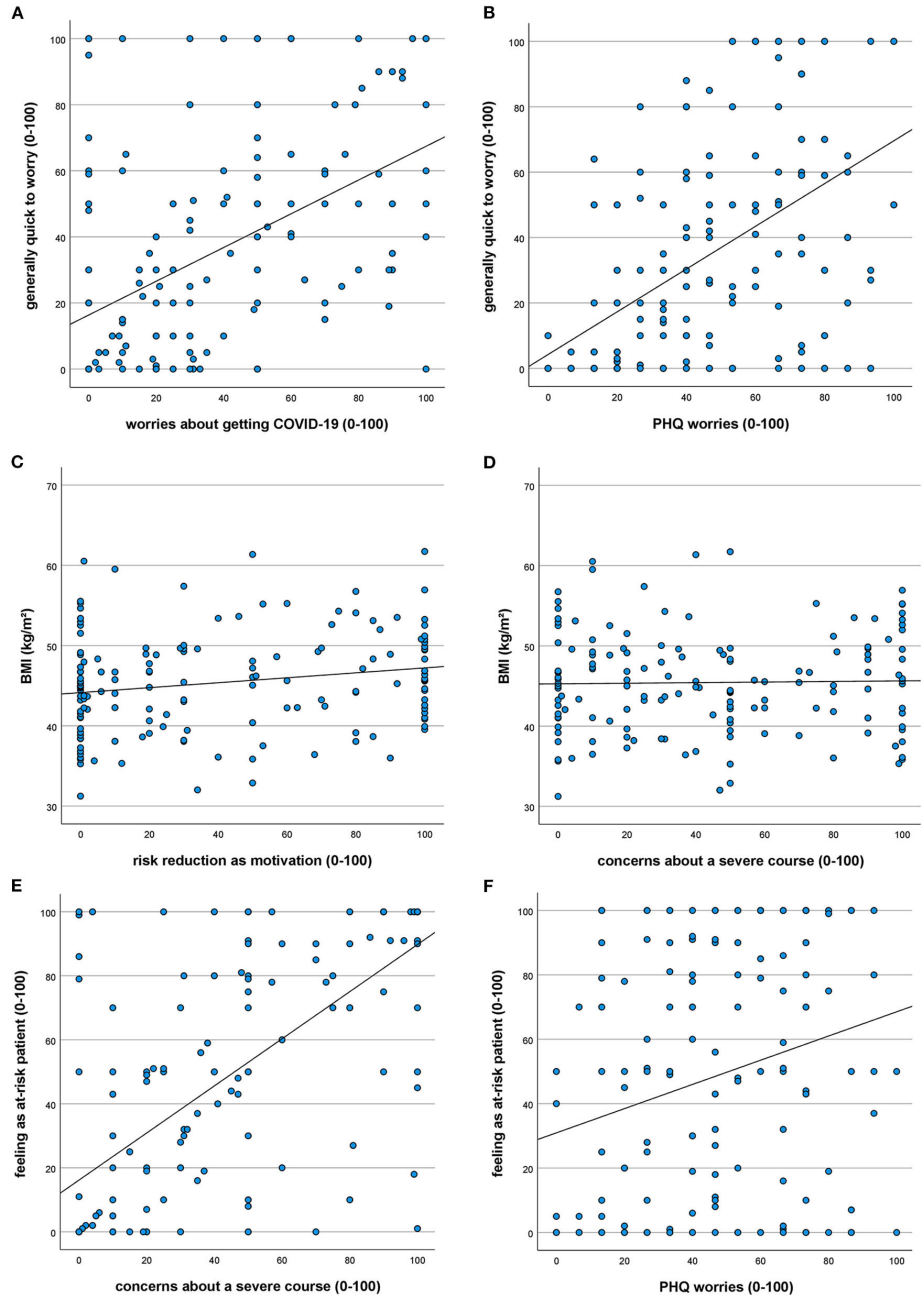
Correlations regarding COVID-19-related concerns. **(A–F)** Data are displayed as scatterplots with fit line.

Overall worries (question 4) correlated significantly with worries about contracting COVID-19 (question 2; *r* = 0.483, *p* < 0.001; Figure 1A) and the worries scale in PSQ (*r* = 0.477, *p* < 0.001; Figure 1B). We found significant positive but small correlation between BMI and motivation to reduce weight (question 7; *r* = 0.195, *p* = 0.016; Figure 1C) but no significant correlations between BMI and COVID-19-related concerns (e.g., question 3; *r* = 0.021, *p* = 0.793; Figure 1D). There was a medium to large significant correlation between feeling like an at-risk patient (question 6) and all questions about anxiety and worries (1–5, *r* = 0.393 to *r* = 0.674, *p* < 0.001; e.g., question 3; *r* = 0.674, *p* < 0.001; Figure 1E) as well as a small significant correlation with worries in PSQ (*r* = 0.237, *p* = 0.003; Figure 1F).

Patients seeking conservative vs. surgical treatment did not differ in COVID-19-related concerns (*t*-test) with two exceptions: Patients asking for bariatric surgery reported significant lower worries to get COVID-19 (question 2; mean ± SD: 33.03 ± 31.21 vs. 48.45 ± 33.07, *p* = 0.019) and lower worries in general (question 4; 31.94 ± 33.34 vs. 46.62 ± 34.15, *p* = 0.035).

### PSQ

**Figure 2:**
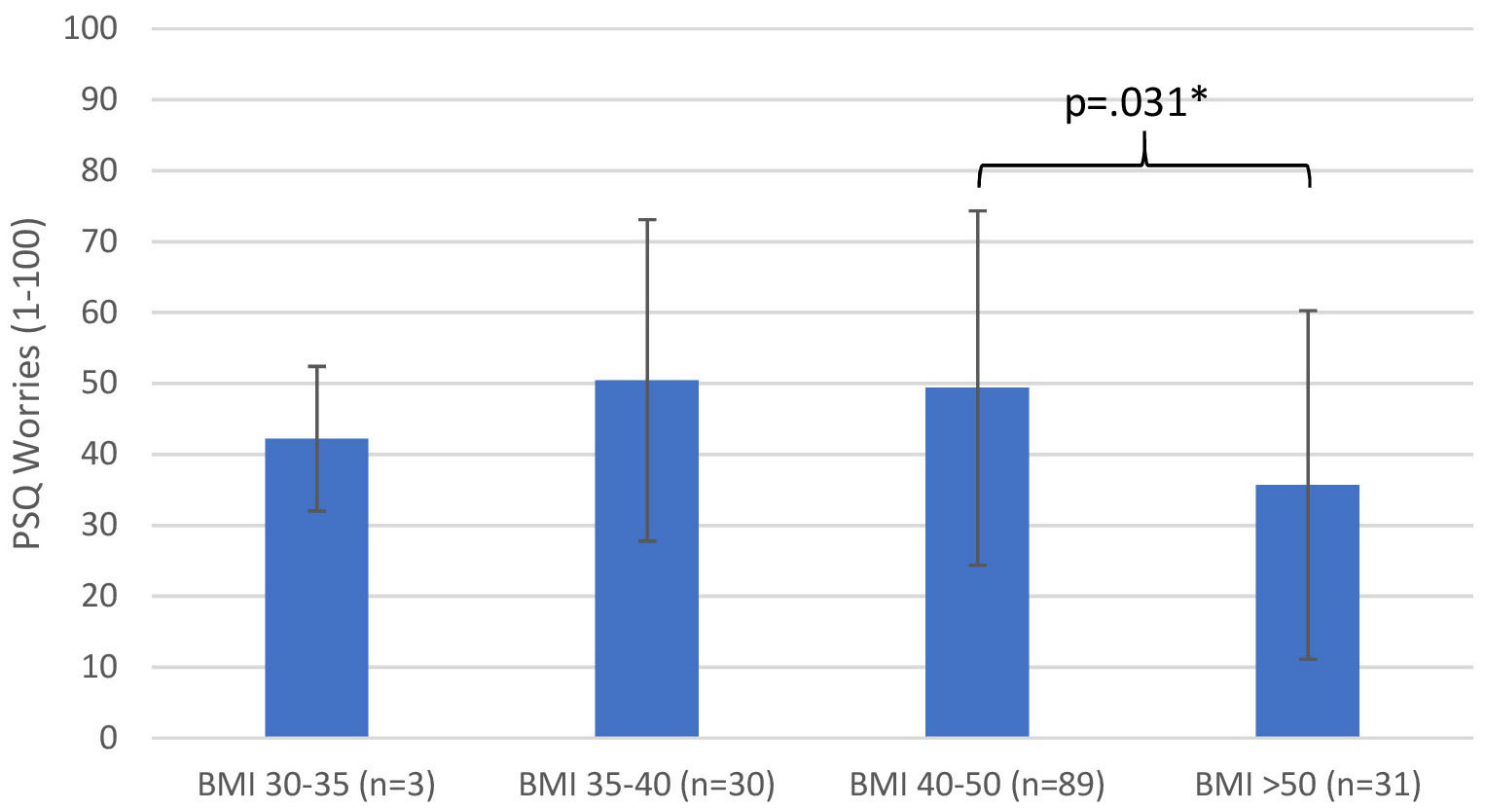
PSQ scale worries. Data are displayed as mean ± SD. ^*^p < 0.05 as assessed by ANOVA and Tukey *post-hoc* test.

A significant effect was found in ANOVA related to the PSQ scale worries and BMI groups [*F*__(3_, 149)_ = 2.74, *p* = 0.046]. The patients with highest BMI (group 4, average value 35.70) reported significantly less worries than group 3 (*p* = 0.031; Figure 2). It should be noted that in the worries scale, BMI group 2 with the value 50.44 and BMI group 3 with the value 49.36 were above the cutoff value of the general population (cutoff value PSQ: worries 6.0–46.0). No significant differences have been found for total PSQ as well as the other subscales tension, joy, and demands among BMI subgroups (see Table 3).

**Table 3:** Perceived stress questionnaire.

**Perceived stress questionnaire**	**Average**	**SD**
**Worries**		
BMI group 1 (*n* = 3)	42.22	10.18
BMI group 2 (*n* = 30)	50.44	22.67
BMI group 3 (*n* = 89)	49.36	25.00
BMI group 4 (*n* = 31)	35.70	24.56
	*F*_(3, 149)_ = 2.739	*p* = 0.046
**Tension**		
BMI group 1 (*n* = 3)	46.67	13.33
BMI group 2 (*n* = 30)	58.22	21.65
BMI group 3 (*n* = 89)	53.63	25.00
BMI group 4 (*n* = 31)	45.81	25.22
	*F*_(3, 149)_ = 1.451	*p* = 0.230
**Joy**		
BMI group 1 (*n* = 3)	37.78	26.94
BMI group 2 (*n* = 30)	44.00	21.63
BMI group 3 (*n* = 89)	45.84	23.43
BMI group 4 (*n* = 31)	52.47	20.78
	*F*_(3, 149)_ = 1.013	*p* = 0.389
**Demands**		
BMI group 1 (*n* = 3)	51.11	13.88
BMI group 2 (*n* = 30)	51.56	23.35
BMI group 3 (*n* = 89)	44.57	22.60
BMI group 4 (*n* = 31)	38.71	18.57
	*F*_(3, 149)_ = 1.836	*p* = 0.143
**PSQ total**		
BMI group 1 (*n* = 3)	50.56	12.29
BMI group 2 (*n* = 30)	54.06	17.87
BMI group 3 (*n* = 89)	50.43	19.75
BMI group 4 (*n* = 31)	41.94	18.50
	*F*_(3, 149)_ = 2.270	*p* = 0.083

*Differences between groups were assessed by ANOVA and Tukey post-hoc test*.

## Discussion

In the present explorative study, we assessed COVID-19-related concerns and the related motivation to reduce body weight in a naturalistic setting in the middle of the pandemic using a population of subjects presenting to an obesity center with the aim to reduce body weight.

Using an *ad hoc* questionnaire, obese patients (BMI range 31.2 to 61.7) reported medium values for the feeling of being at-risk for a severe course of a COVID-19 disease. On the other hand, they reported values above average (59.66, 0–100) in feeling sufficiently informed with regard to COVID-19. The motivation to reduce weight because of thereby reducing the risk of getting severely ill with COVID-19 was scored at below medium levels. Interestingly, COVID-19-related concerns and BMI did not show a correlation, giving rise to the assumption that increasing BMI does not increase the perception/awareness of being at higher risk for a severe course of the COVID-19 disease. In addition, when clustering patients according to BMI groups, the group with the highest BMI (>50) even showed decreased levels on the worries scale of the Perceived Stress Questionnaire.

Although the risk factor obesity for a severe course of the COVID-19 disease is well known to medical professionals (7, 8, 16), this might not be true for the lay public. How can we communicate an elevated risk to at-risk patients in another way with encouraging character that helps them to care for themselves? Communicating worries and stirring up fears do not seem to work properly and may have unwanted side effects like stigmatization (17). In particular, obese patients who experienced an inability to reduce weight on their own in the past could be in danger of giving up and denying the risk. This can be exacerbated by continuing to communicate only the risk, which can be overwhelming and forcing a respective denial. Therefore, the motivation to reduce weight should be separated from the communication of an increased risk due to obesity and should not be used as an argument when accompanying decision-making. When communication about a risk is combined with a choice of viable alternative actions to reduce it, self-efficacy can be increased—an important motivator for behavior change (18). Another reason for the observed low levels of worries in the group of highest BMI could be anxious components, which lead to increased apathy and less concern about contracting the virus. We did not measure anxiety in our study, which could be an interesting parameter for further studies.

One major strength of the study is that it has been conducted in the middle of the COVID-19 pandemic. Because of this and its naturalistic design, we think that it can contribute to the considerations on how obese patients could be motivated when communicating new risk information. Restrictions are the cross-sectional and self-reported design. As we only used a narrow psychometric test with PSQ, there could be confounders in our data that we did not detect and therefore cannot explain. Because of the explorative manner, the small sample size, and small correlations, our study cannot be generalized to the average population. Furthermore, it is limited to the assessed geographic region.

## Conclusion

Our findings suggest that risk communication for obese patients in the context of COVID-19 did not work very well for all patients, especially for individuals with very high BMI (>50). We did not find a strengthened wish for reducing weight to reduce the risk for a severe course of COVID-19. Future studies in this area should focus on post-COVID-19 times and try to examine how risk perception of people with obesity changed. This could also have an impact on the desire for bariatric surgery or conservative treatment. They could also focus on the aspect of how to motivate obese people for weight reduction in the context of communicating risks for sequelae and take possible moderating demographic variables into account.

## Data Availability Statement

The raw data supporting the conclusions of this article will be made available by the authors, without undue reservation.

## Ethics Statement

The studies involving human participants were reviewed and approved by Ethics Committee of the University Hospital Tuebingen. The patients/participants provided their written informed consent to participate in this study.

## Author Contributions

NS and JL performed the data analysis and wrote the first draft of the manuscript. AS, NS, RA, IM, and SZ planned and initiated the study. AS conducted the data generation and gave critical input throughout the study. All authors finalized the manuscript.

## Conflict of Interest

The authors declare that the research was conducted in the absence of any commercial or financial relationships that could be construed as a potential conflict of interest.

## Publisher's Note

All claims expressed in this article are solely those of the authors and do not necessarily represent those of their affiliated organizations, or those of the publisher, the editors and the reviewers. Any product that may be evaluated in this article, or claim that may be made by its manufacturer, is not guaranteed or endorsed by the publisher.
